# Supplementary material for: Identification of evolutionarily stable functional and immunogenic sites across the SARS-CoV-2 proteome and greater coronavirus family
Source: Bioinformatics. 2021 May 27;37(22):4033–40. doi: 10.1093/bioinformatics/btab406 (PMC8243408; doi:10.1093/bioinformatics/btab406)
Supplement: btab406_Supplementary_Data [file btab406_supplementary_data.zip › ET_CoV2_R2_SI_v6_no_track_reduced.pdf]

Supporting information for

Identification of evolutionarily stable functional and immunogenic sites across the SARS-CoV-2 proteome and greater coronavirus family

Chen Wang[1], Daniel M. Konecki[1], David C. Marciano [1,]*, Harikumar Govindarajan, Amanda M. Williams, Brigitta Wastuwidyaningtyas, Thomas Bourquard, Panagiotis Katsonis and Olivier Lichtarge*

[1]These authors contributed equally

*Correspondence: david.marciano@bcm.edu (D.C.M), lichtarge@bcm.edu (O.L.)

This section includes:

Supporting materials and methods, pages 2-5

Table S1, page 6

Figures S1 to S8, pages 7-15

Legends for Datasets S1 to S9, pages 16

SI References, pages 17-19

Other supplementary materials for this manuscript include the following:

Dataset S1. Alignment Sequence Counts Each Filtering Step.

Dataset S2. ET Rankings and Unique Variant Counts.

Dataset S3. SCW Z-scores.

Dataset S4. Enrichment of important ET residues in ligand binding sites

Dataset S5. Structural Sites with 5A Proximity Cutoff.

Dataset S6. Linear Sites.

Dataset S7. Comparison of Linear ET Sites and Structural ET Sites.

Dataset S8. Cross-reactive T Cell Epitopes Training Set.

Dataset S9. Cross-reactive T cell Epitopes Prediction Set.

SI Materials and Methods:

Reference Sequence Retrieval and Variant Analysis of the Current SARS-CoV-2 Outbreak

SARS-CoV-2 isolate Wuhan-Hu-1 was used as the reference strain. Its genomic and proteomic sequences were downloaded from GenBank (NC_045512.2). ORF1a and ORF1ab were broken down into NSPs according to the ranges described in the genbank file.

Sequences of SARS-CoV-2 clinical strains were downloaded from the GISAID (Shu and McCauley, 2017), Genbank (Benson *et al.*, 2018) and the China National Center for Bioinformation (CNCB) (Zhao *et al.*, 2020) on December 8th, 2020. We obtained 139607 SARS-CoV-2 sequences after filtering out incomplete and low-quality ones according to the meta data from CNCB. These sequences were then aligned to Wuhan-Hu-1 using minimap2 (Li, 2018). Single nucleotide variants were called using bcftools (Li, 2011) and then converted into amino acid variants with an internal R script. The number of unique amino acid variants at each position was calculated.

Homolog Sequence Retrieval and Alignment

Databases: The UniRef90 and UniRef100 sequence databases were downloaded from https://www.uniprot.org/downloads (on 5/12/2020 and 5/15/2020 respectively)(Bateman, 2019) and were subsequently filtered to remove any sequences which contained the terms "Fragment" or "LOW QUALITY". The resulting fasta files were used to make databases for the BLAST+ tool (Camacho *et al.*, 2009) using the makeblastdb utility. The NCBI NR BLAST+ database was downloaded through the update_blastdb.pl utility on 5/12/2020.

BLAST: Homologs were identified by using the blastp (version 2.9.0, build May 27 2019) tool to search the three databases described above for each of the reference sequences described in the previous section. Settings used for blastp were a max e-value of 0.05 (Mihalek *et al.*, 2004) and maximum number of target sequences 20,000.

Filtering and Alignment: Sequences returned by each BLAST search were iteratively filtered and aligned. The first filtering step removed any sequences with less 70% coverage of the query sequence, or where the query sequence has coverage <70% of the returned sequence. Additional filtering was performed to remove any sequences whose identity with respect to the query sequence were < 25% or >98% (Lichtarge *et al.*, 2002; Mihalek *et al.*, 2004). Sequences containing any amino acid besides the standard twenty amino acids or gap symbol were also removed. Finally, sequence descriptions containing the words "artificial", "fragment", "low quality", "partial", or "synthetic" and sequences whose taxonomy included the words "synthetic" or "artificial" were also removed. The sequences passing these filters were aligned using ClustalW (version 2.1) (Larkin *et al.*, 2007; Madabushi *et al.*, 2002)with the align and quick tree options set to True. The alignment is filtered again by computing all pairwise sequence identities and keeping only one sequence from any group with sequence identity > 98%. All sequences passing this second filter had the gaps from the first alignment removed and were aligned again using ClustalW, with the same settings specified above, to create final alignments.

Sequence counts are available for all final alignments (**Figure S1, Dataset S1**), only two proteins had too few matches for ET, NSP11 and ORF10, both of which have unknown function and very short reference sequences (13 and 38 amino acids, respectively), while most other nonstructural proteins, the membrane protein and the nucleocapsid protein each returned at least 50 sequences.

Structure specific analyses for NSP3 domains were performed by using the amino acid sequence of a specific PDB structure and a specific chain in place of the Wuhan-HU-1 reference sequence. All other aspects of homolog retrieval and alignment were performed as described above. The size of the resulting alignments can be seen in **Figure S5A**.

Evolutionary Trace

Evolutionary Trace was run for each protein for each of the three alignments produced using the method described above. Residue importance was computed using the rvET method (Mihalek *et al.*, 2004), which is made publicly available through the UET server (Lua *et al.*, 2016). Briefly, this method constructs a UPGMA like tree using the BLOSUM62 distance matrix for a given alignment. The trees constructed for most nonstructural proteins, the membrane protein and the nucleocapsid protein each span the Alpha, Beta, Delta, and Gamma genera of coronavirus (**Supplemental Figures 1-4**). NSP1, NSP2, NSP3, the Spike protein (S), the envelope protein (E) and the remaining accessory proteins, each consisted almost exclusively of betacoronavirus homologs using our sequence identify cutoffs. These results suggest some coronavirus proteins (NSP1-3, S, E and the accessory proteins) are diverging more rapidly or were recruited at a different point than the core viral proteins involved in viral RNA synthesis, modification and packaging. These trees along with Shannon entropy are used to measure how patterns of invariance correspond with phylogenetic divergence following the formula:

$$\rho_i = 1 + \sum_{n=1}^{N} \frac{1}{n} \sum_{g=1}^{n} \left(- \sum_{a=1}^{20} f_{ia}^{g} \ln\left(f_{ia}^{g}\right) \right)$$

Where ρ_i is the rank of residue i, N is the height of the phylogenetic tree, n is a specific level in the tree (1 being the root and N being the leaves), g is a specific branch at level n, and a is an amino acid. The ET ranking of a residue position is the percentile ranking (0-100, 0 for most evolutionarily important) of its raw ET score. An average trace was also computed by taking the raw residue importance scores from each of the three traces performed, averaging them and computing a new ET ranking based on the averaged raw scores.

Evolutionary Action

Evolutionary Action (EA) was calculated for each coding mutation for every SARS-CoV-2 protein using previously described method (Katsonis and Lichtarge, 2014). In brief, EA models the genotype-to-phenotype relationship formally as: $f(\gamma) = \varphi$, where γ is the genotype, φ is the fitness phenotype, and f is the evolutionary fitness function. When a mutation occurs at position i from amino acid X to Y, denotes as $r_{i,X\to Y}$, EA is defined as:

$$EA \equiv \Delta\varphi \approx \frac{\partial f}{\partial r_i} \cdot \Delta r_{i,X\to Y}$$

Where $\partial f/\partial r_i$ is the evolutionary fitness gradient, and $\Delta r_{i,X\to Y}$ is the genotype perturbation of that mutation. $\partial f/\partial r_i$ is approximated using the ET scores determined in this study. $\Delta r_{i,X\to Y}$ is approximated by the substitution odds. Then the EA scores of every single amino acid within a

protein are normalized to 0-100, with 0 and 100 as the least and the most evolutionarily impactful mutation.

Selection Cluster Weighting (SCW) Z-Score Evaluation

Traces were evaluated using the unbiased form of the SCW z-score metric (Mihalek *et al.*, 2004, 2007; Wilkins *et al.*, 2010, 2013; Madabushi *et al.*, 2002), which measures how clustered top ranked residues are on the three-dimensional protein structure, it is available through the PyETViewer plugin for PyMol (Lua and Lichtarge, 2010). Briefly, the weight assigned to a selection of residues is given by:

$$w = \sum_{i<j}^{L} S(i)S(j)A(i,j)$$

where L is the full set of pairs of residues in a protein (counted only once per pair as specified by the term $i < j$), S is a selection function and returns 1 for a given residue (i or j) if that residue passes a given ET ranking (coverage) cutoff, and A is an adjacency matrix for all residues in the structure where position i,j is 1 if the shortest atomic distance between the two residues is < 4Å and 0 otherwise. For all measurements taken in this manuscript, a cutoff of coverage of ≤ 30 was used for S, meaning that only the top 30% of residues by ET ranking are considered. This cutoff has been used in previous work (Wilkins *et al.*, 2010, 2013) and has been characterized as the point where clustering loses significance (Madabushi *et al.*, 2002).

Enrichment of important residues in ligand binding sites

In order to compare our approach with a more general percent identity approach by Gupta et. al (Gupta *et al.*, 2020) in predicting evolutionary important residues in SARS-CoV-2 proteins, we scaled the conservation scores reported by Gupta et. al to 0-100, with 0 as the most conserved residue. Protein surface atoms (accessible surface area >= 0.04 $Å^2$) within 5 Å of biological ligands were defined as binding atoms. A binding atom was considered evolutionarily important by ET or Gupta's results if its residue's ET ranking or scaled conservation score was <= 30. One-sided Fisher exact test was performed to test the enrichment of important surface atoms (evaluated by ET or by Gupta et.al) at the biological binding sites.

Evolutionary Therapeutic Sites

Structural sites were predicted by filtering all atoms in a protein structure for the Evolutionary Trace coverage score and unique variant count of their residue, and their surface accessibility. An atom was considered a candidate if its residue's ET ranking was <= 30 and had 0 unique SARS-CoV-2 variants (i.e. has not been seen to vary in the current outbreak), and if it had an accessible surface area >= 0.04 $Å^2$ as measured by the get_area command in PyMol (The PyMOL Molecular Graphics System). The distance between candidate atoms was then measured and clusters of atoms within 5 angstroms of one another were formed. For each such cluster the residue to which each atom belongs was identified and clusters with >2 residues were nominated as potential structural epitopes.

Linear epitopes were defined as consecutive amino acids (more than 1) in the protein sequences that have ET ranking <= 30 and that are not mutated in the current outbreak.

Comparison to Known Drugs

Structural epitopes (5Å cutoff) were identified using apo form structures, then mapped to the co-structures of NSP3 with peptide inhibitor vir251 (PDB:6wx4), NSP5 with potential drug 13b (PDB:6y2f), NSP12 with drug remdesivir (7bv2), NSP15 in complex with potential drug tipiracil (PDB:6wxc), and NSP16 with sinefungin (PDB:6wkq). Remdesivir has been shown to speed up the recovery of COVID-19 patients in clinical trials (Beigel *et al.*, 2020), while the α-ketoamide inhibitor 13b can suppress SARS-CoV-2 replication in cell lines (Zhang *et al.*, 2020). Vir251 and tipiracil were also shown to effectively inhibit the enzymatic activities of their targets (Rut *et al.*, 2020; Youngchang Kim *et al.*, 2020). The remaining drug, sinefungin, is a pan-MTnase (NSP16) inhibitor that inhibits the growth of yeast cells ectopically expressing NSP16 from SARS-CoV (Decroly *et al.*, 2011).

Analysis of Cross Reactive Epitopes

Mateus et al. identified 61 SARS-CoV-2 T-cell epitopes (15mers) that have homologous sequences in other Alphacoronaviruses and Embecoviruses (subgenus of Betacoronaviruses), which include 4 common human coronaviruses (229E, NL63, HKU1 and OC43) (Mateus *et al.*, 2020). 40 of those epitopes were tested for cross reactivity against their homologs, which we used as a training set. The homologs of the remaining 21 SARS-CoV-2 epitopes that weren't tested for cross-reactivity were used as a testing set. The Evolutionary Action (EA), which predicts the impact of a given amino acid substitution in a protein, for each SARS-CoV-2 protein was calculated based on previously described method (Katsonis and Lichtarge, 2014). For each homolog in the training set, the percent identity, sum of (100 - ET rankings), sum of EA scores for mutated residues (sumEA), and sumEA/sum(100 - ET rankings) were calculated. Note that sum of (100 - ET rankings) was calculated for all residues in the 15 mer, while the sumEA was only based on the mutated positions. The optimal cut point (0.168) of sumEA/sum(100 - ET rankings) that best separates cross reactive and not cross reactive homologs was determined by maximizing the F1 score. The optimal cut point was then applied to the testing set to predict cross reactivity.

Table S1. List of structures used in main figures:

Protein(s)	PDB	Reference
NSP1	7k7p	(Clark *et al.*, 2020)
NSP1	6zlw	(Thoms *et al.*, 2020)
NSP3	6woj	(Alhammad *et al.*, 2020)
NSP3	6w9c	(Osipiuk *et al.*, 2020)
NSP3	6wx4	(Rut *et al.*, 2020)
NSP4	ExPasy NSP4 model 01	(Bienert *et al.*, 2017; Waterhouse *et al.*, 2018; Studer *et al.*, 2020; Non-structural protein 4 (nsp4) | P0DTD1 PRO_0000449622 | Models)
NSP5	6yb7	(Owen *et al.*, 2020)
NSP5	6y2f	(Zhang *et al.*, 2020)
NSP9	6wxd	(Littler *et al.*, 2020)
NSP7, 8, 12, and 13	6xez	(Chen *et al.*, 2020)
NSP12	7bv1	(Yin *et al.*, 2020)
NSP12	7bv2	(Yin *et al.*, 2020)
NSP12	6xez	(Liu *et al.*, 2020)
NSP13	6zsl	(Newman *et al.*, 2020)
NSP10 and 14	5c8s	(Ma *et al.*, 2015)
NSP15	6wlc	(Y. Kim *et al.*, 2020)
NSP15	6wxc	(Youngchang Kim *et al.*, 2020)
NSP15	6x1b	(Youngchang Kim *et al.*, 2020)
NSP10 and 16	6w4h	(Minasov, Shuvalova, Rosas-Lemus, Kiryukhina, Wiersum, *et al.*, 2020)
NSP16	6wkq	(Minasov, Shuvalova, Rosas-Lemus, Kiryukhina, Satchell, *et al.*, 2020)
NSP16	6wvn	(Rosas-Lemus *et al.*, 2020)
S	6vsb	(Wrapp *et al.*, 2020)
S	6vsb_1_1_1	(Woo *et al.*, 2020)
ORF3a	6xdc	(Kern *et al.*, 2020)
E	5x29	(Surya *et al.*, 2018)
ORF7a	6w37	(Nelson *et al.*, 2020)
N	6vyo	(Chang *et al.*, 2020)
N	6zco	(Zinzula *et al.*, 2020)

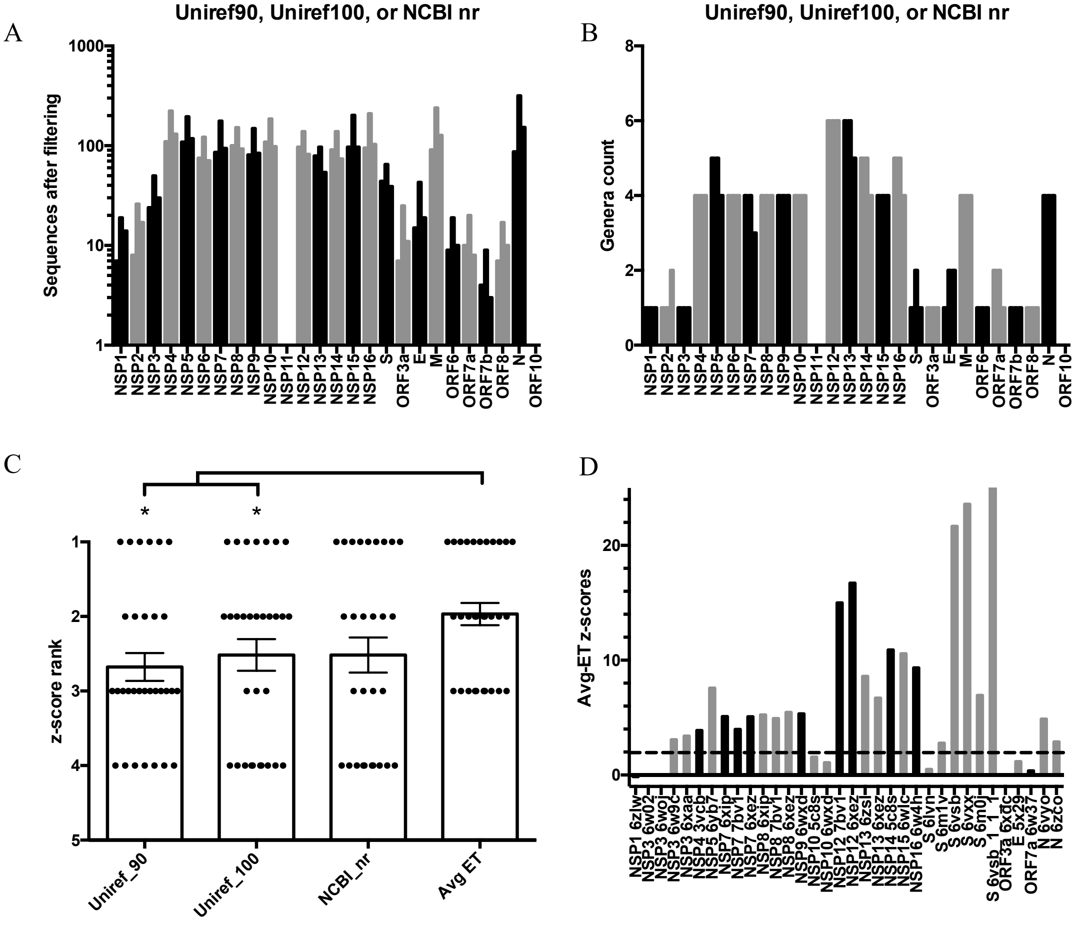

Supplementary Figure 1. Quality of homologous sequences and ET. A) Number of homologs used for each SARS-CoV-2 protein to build the respective multiple sequence alignment. Bars corresponding to neighboring proteins along the genome are shown in alternating black and gray colors. B) Number of coronavirus genera covered by the homolog selections. Genera count = >4 indicates evolutionary information was gathered beyond the Orthocoronavirinae subfamily. C) The z-score rankings for using different protein sequence databases. * indicates significant difference (<0.05, paired t-test, n=31) compared to z-score rankings of average ET scores. D) The distribution of clustering z-scores for each protein using average ET scores. Bars corresponding to neighboring proteins along the *x*-axis are shown in alternating black and gray colors. The dashed line corresponds to a z-score of 2, above which is considered significant.

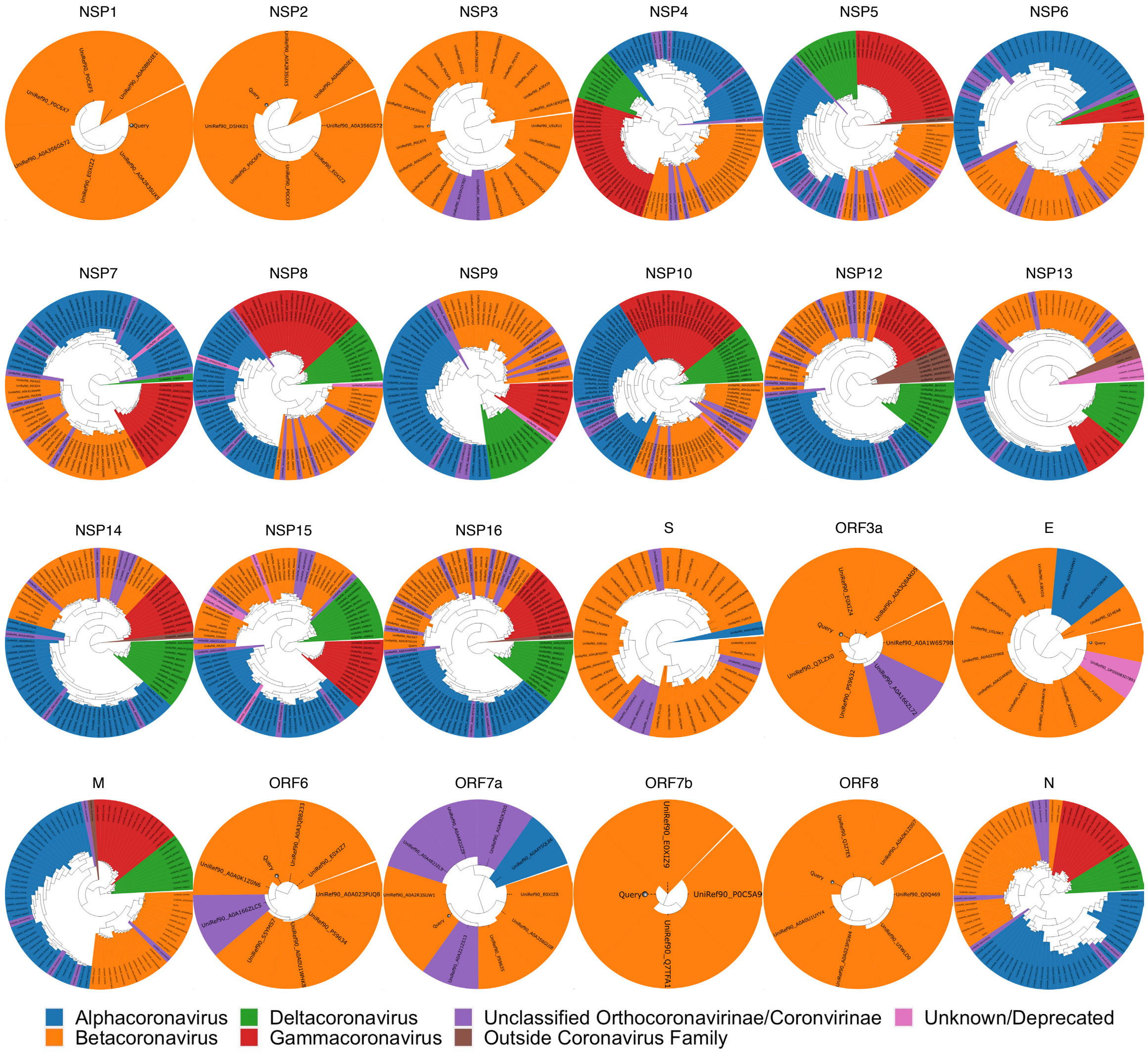

Supplementary Figure 2. Phylogenetic trees generated using the sequences recovered from the UniRef90 sequence database.

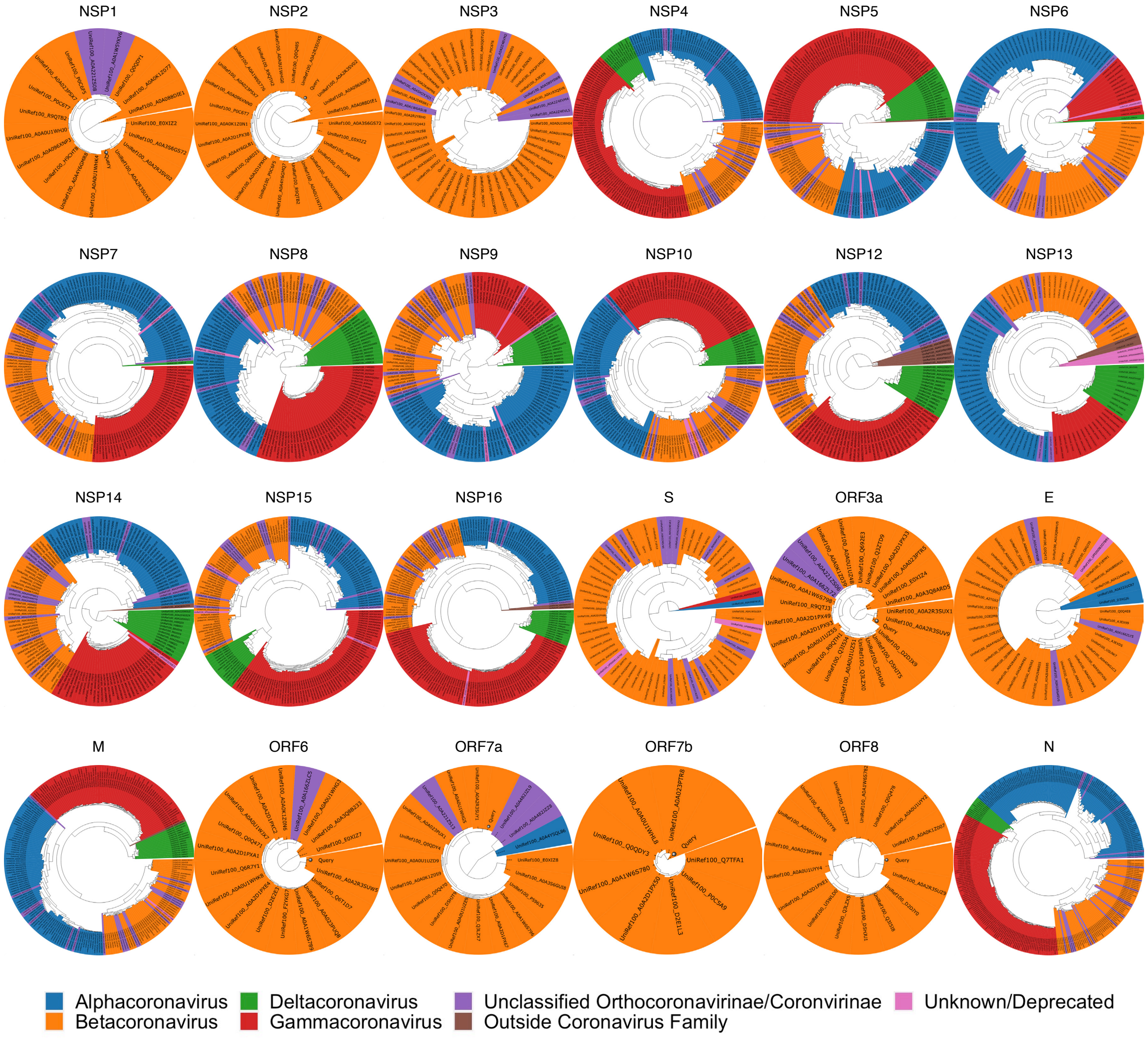

Supplementary Figure 3. Phylogenetic trees generated using the sequences recovered from the UniRef100 sequence database.

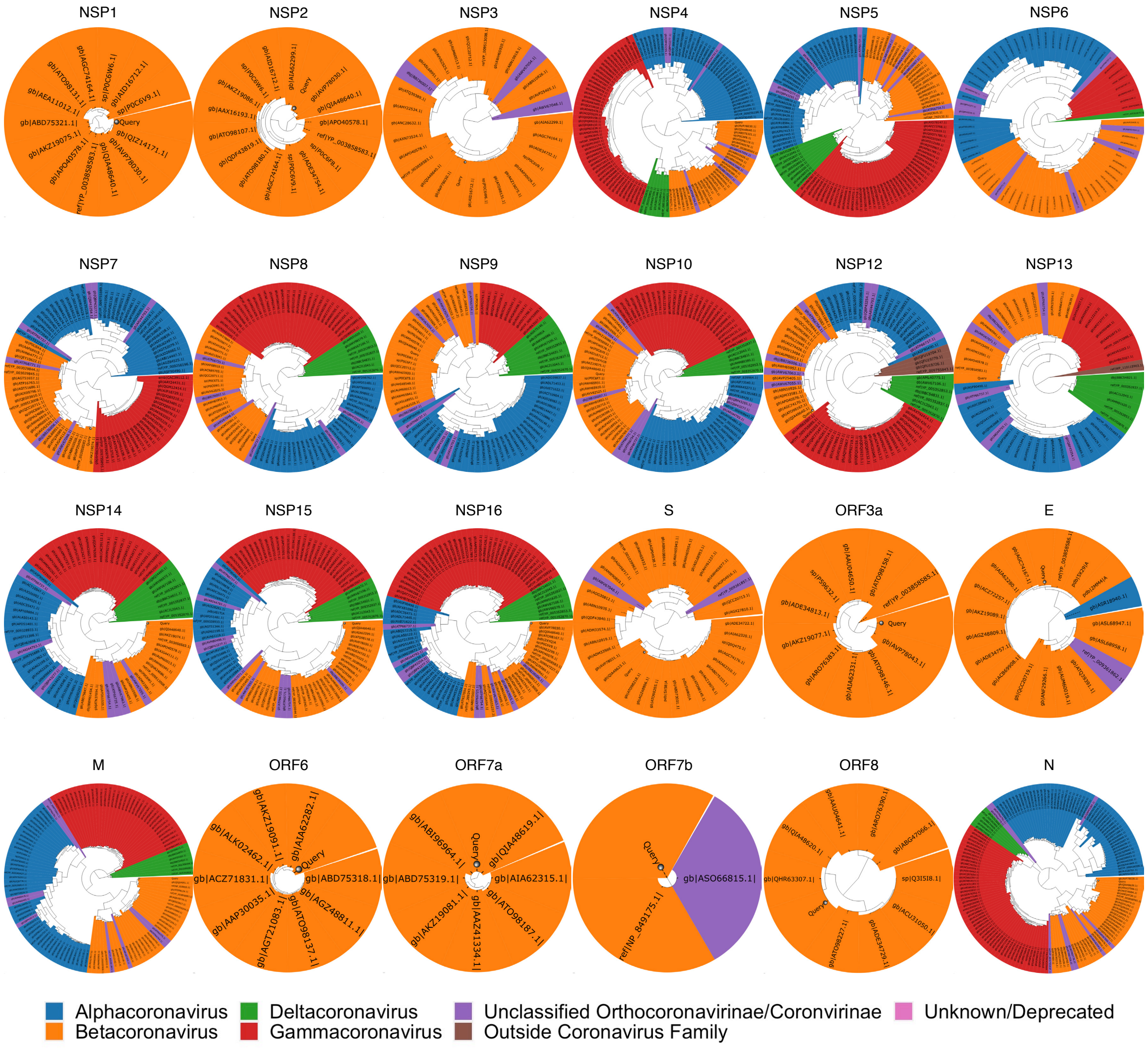

Supplementary Figure 4. Phylogenetic trees generated using the sequences recovered from the NCBI NR sequence database.

A

Protein Name	Structure	Chain	U90	U100	NR
NSP3	-	-	24	50	30
NSP3	6w02	A	5695	6180	2705
NSP3	6woj	A	5513	5819	2572
NSP3	6w9c	A	40	76	42

C

Query Sequence	Domain	Structure	Chain	U90	U100	NR	Averaged
NSP3	ADP Ribose Phosphatase	6w02	A	-	-	-	-
NSP3	Macrodomain	6woj	A	-	-	-	-
NSP3	PL2Pro	6w9c	A	2.64	2.551	3.502	3.082
NSP3	Pl2Pro + Ubiquitin Propargylamide (human)	6xaa	A	3.178	2.957	3.819	3.387
6w02A	ADP Ribose Phosphatase	6w02	A	7.954	8.848	8.55	9.594
6wojA	Macrodomain	6woj	A	9.286	8.69	9.436	9.585
6w9cA	PL2Pro	6w9c	A	5.521	4.672	4.279	5.645
6w9cA	Pl2Pro + Ubiquitin Propargylamide (human)	6xaa	A	5.537	5.012	5.136	6.072

B

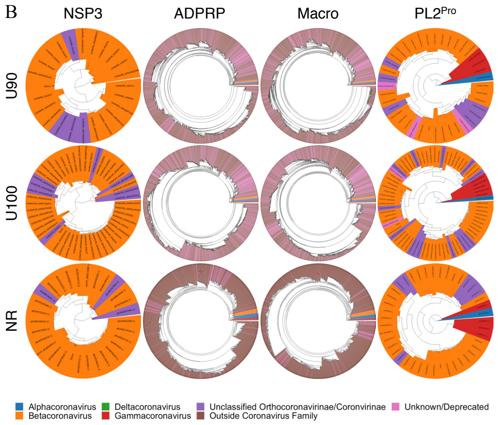

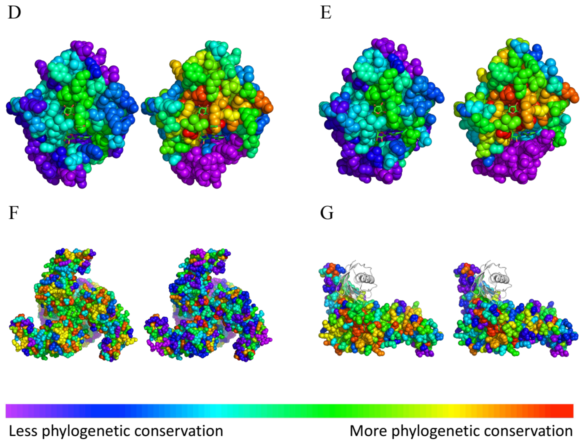

Supplementary Figure 5. Performing sequence selection and Evolutionary Traces for the full NSP3 reference sequence and for specific structures/domains leads to dramatic changes in the recovered sequences and identification of key residues. A) Number of sequences retrieved when indicated databases queried with the full NSP3 reference ('-' indicates no full-length structure or corresponding chain), ADP-ribose-phosphatase domain sequence (6w02), Macro domain (6woj) or the papain-like protease domain (6w9c). B) Phylogenetic trees generated for alignments in A showing the wide coverage of alignments for the ADPRP subdomain and macro domain and the much narrower coverage of phylogeny in the PL2^{pro} domain and full reference sequence. C)SCW Z-Scores measured for NSP3 structures for Evolutionary Traces resulting from the full reference (NSP3) and with queries based on specific structures and chains. D-G) The Evolutionary Trace scores mapped onto apo NSP3 structures 6w02 chain A (D), 6woj chain A (E), 6w9c (F), and 6xaa (G) with the full NSP3 reference scores on the left and the structure specific query on the right. G) Phylogenetic trees of sequences retrieved from indicated databases using sequence from full-length NSP3, the ADP-ribose-phosphatase domain (ADPRP), the Macro domain or, the papain-like protease domain (PL2^{Pro}).

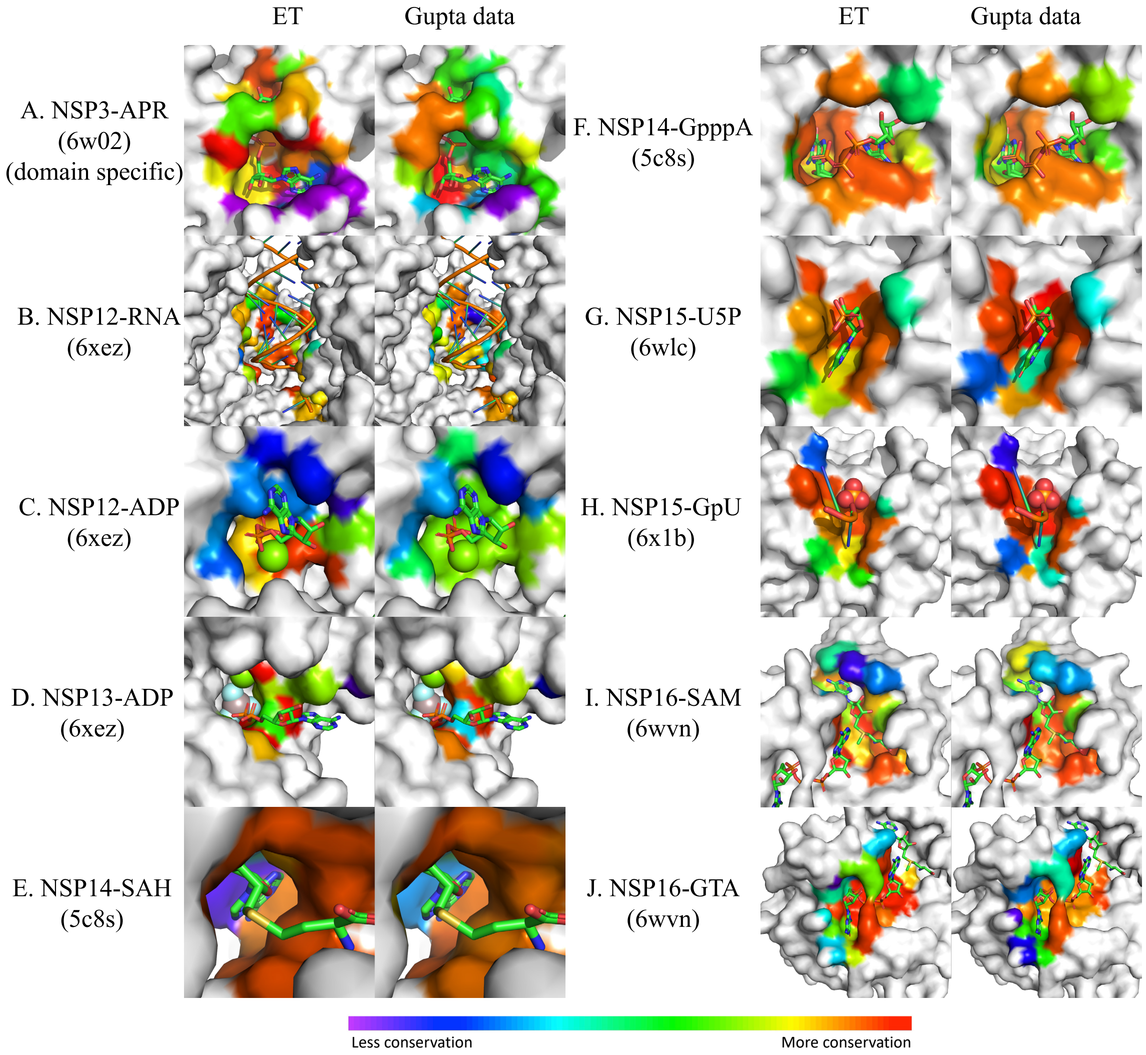

Supplementary Figure 6. Conservation measurements of residues at the ligand binding sites for different SARS-CoV-2 proteins. ET was compared with conservation measurement reported by Gupta et. al. The conservation scores reported by Gupta et. al were scaled to 0-100, with 0 as the most important/conserved, in order to directly compare to ET. Surface atoms within 5 Å of the ligands are colored by their ET or scaled conservation scores. ET and conservation scores were calculated based on entire protein, except in the NSP3- adenosine-5-diphosphoribose (APR) complex the domain specific scores were used. (A) ADP ribose phosphatase domain of NSP3 with APR. (B) NSP12 with RNA. (C) NSP12 with ADP (adenosine-5'-diphosphate). (D) NSP13 (helicase) with ADP. (E) NSP14 (guanine-N7 methyltransferase) with SAH (S-adenosyl-L-homocysteine). (F) NSP14 with GpppA (guanosine-P3-adenosine-5',5'-triphosphate). (G) NSP15 (uridylate-specific endoribonuclease) with U5P (uridine-5'-monophosphate). (H) NSP15 with Nucleotide GpU. (I) NSP16 (2'-O-methyltransferase) with SAM (S-adenosylmethionine). (J) NSP16 with GTA (P1-7-methylguanosine-P3-adenosine-5',5'-triphosphate).

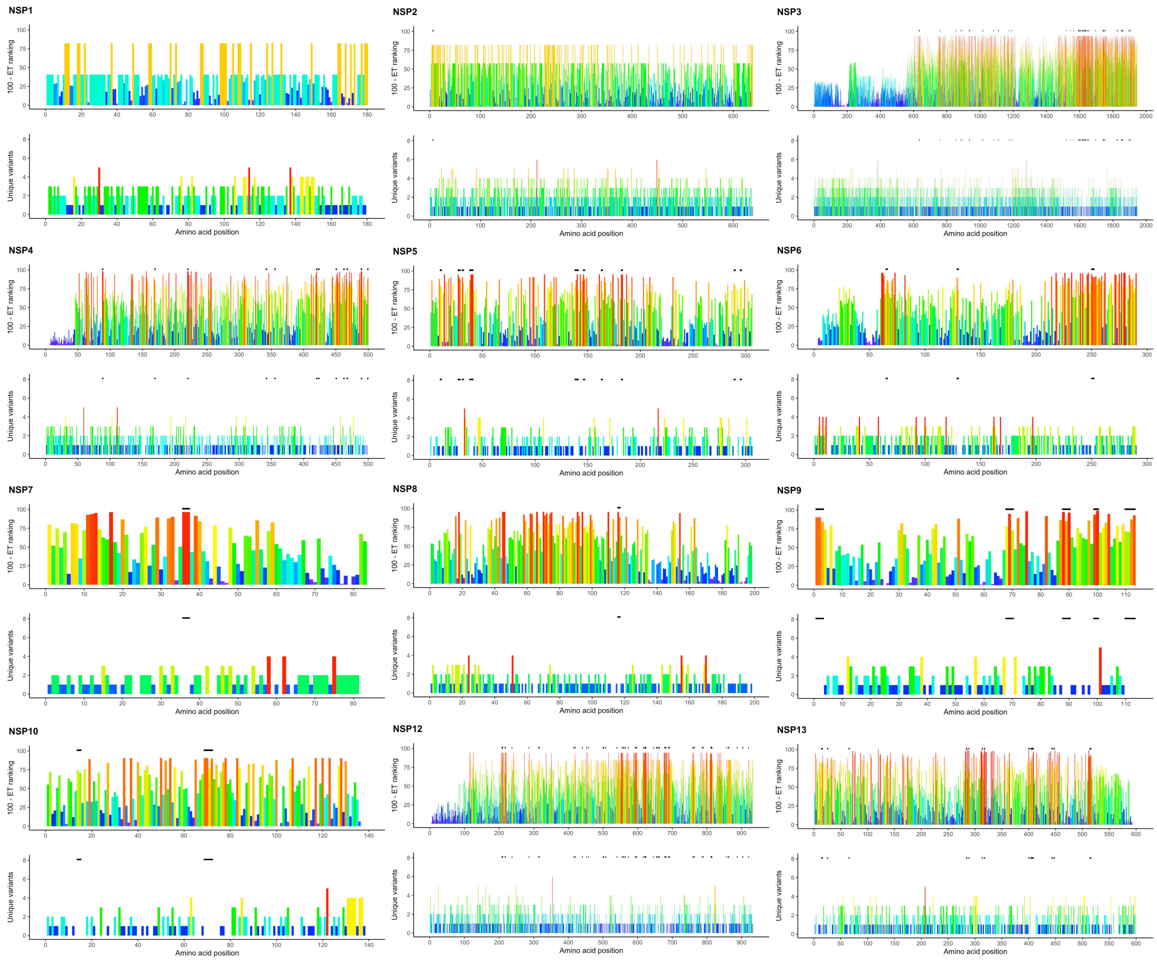

Supplementary Figure 7 (continued, next page). Identification of linear epitopes from coronavirus family combining evolutionary information from the coronavirus family with the current outbreak. Identification of linear epitopes in each SARS-CoV-2 protein with an Evolutionary Trace. Past evolutionary information is represented by 100 - ET ranking. Number of unique variants summarizes the evolutionary information in the current outbreak. Tandem regions in the linear sequence with low ET ranking (<= 30) and no current mutations are shown as blacklines above each plot.

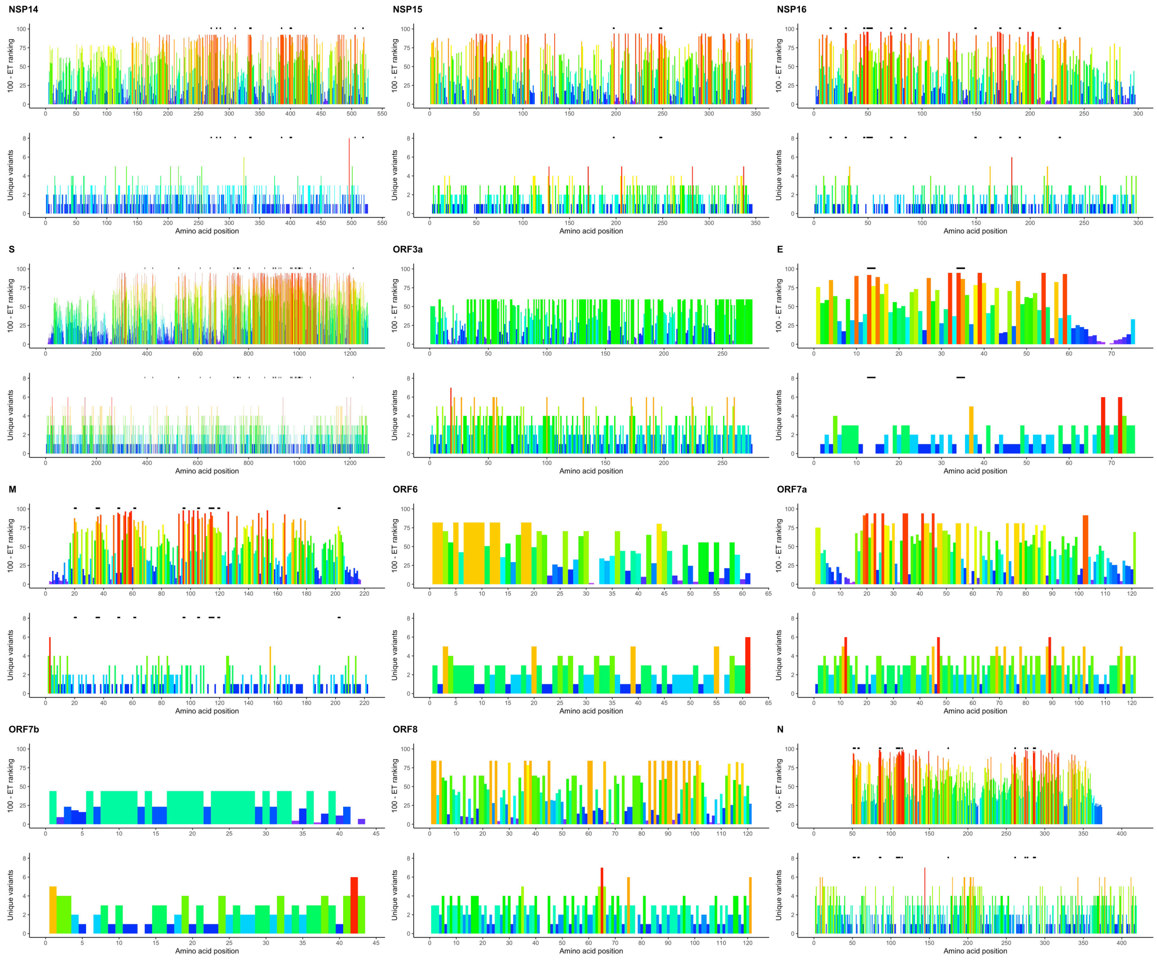

Supplementary Figure 7 (continued). Identification of linear epitopes from coronavirus family combining evolutionary information from the coronavirus family with the current outbreak.

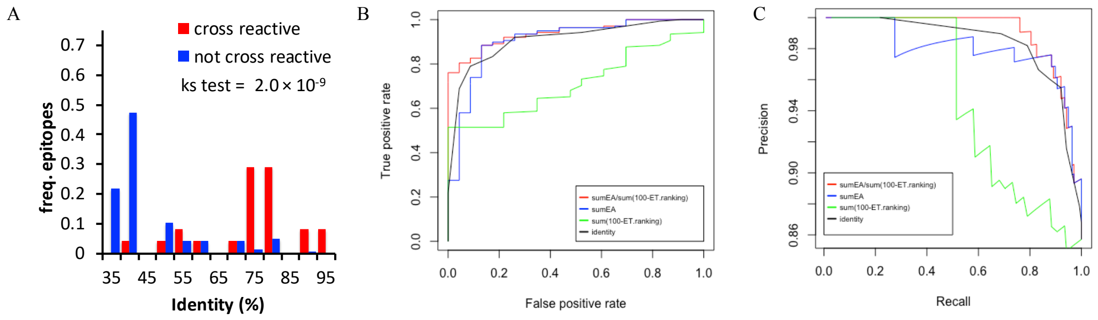

Supplemental Figure 8. The EA/ET metric outperforms several alternative methods to separate cross reactive T-cell epitopes from non-cross reactive epitopes. A) Relative frequency distributions of percent identity (relative to SARS-CoV-2) for T-cell epitopes shown to either be cross reactive (red) or not (blue). A Kolmogorov-Smirnov test (ks test) shows a significant difference in the distributions. B) Receiver operator curves of correctly classifying cross reactive epitopes are shown for EA/ET (sumEA/sum(100-ET ranking)) (red), sumEA (blue), sum(100-ET ranking) (green) and percent identity (black). C) Precision recall curves for the same four classifier approaches.

Legends for Datasets

Dataset S1. Alignment Sequence Counts Each Filtering Step. Reported are the number of sequences from: BLAST hits retrieved from UniRef90, UniRef100 or NCBI non-redundant databases, filtering for our search criteria and second filtering step after removing alignment gaps. Final filtered sequence number data were used to generate Figure S1A.

Dataset S2. ET Rankings and Unique Variant Counts. For each protein, amino acid position, amino acid, ET ranking, 100-ET ranking and number of unique variants in current SARS-CoV-2 outbreak are reported.

Dataset S3. SCW Z-scores. Data for each of the structures used in this study including the selected chain, species of origin, identity to the reference protein sequence, and coverage of the reference are reported. In addition, ET rankings produced by alignments built from the UniRef90, UniRef100, and NCBI NR databases and the average of these rankings are scored using the SCW z-score to determine clustering on each 3D structure. Residues corresponding to ET rankings <= 30 were scored to produce these z-scores.

Dataset S4. Enrichment of important ET residues in ligand binding sites. Data used in, and results of, calculating statistical enrichment (one-sided Fisher's exact test) for highly ranked residues (£30 percentile) within 5 Å of ligands. Protein name, ligand, PDB file name, PDB chain used, total number of surface atoms used in calculation, number of surface atoms within 5 Å of ligands, total number of surface atoms in residues ranked by ET in £30 percentile, number of surface atoms in residues ranked by ET in £30 percentile within 5 Å of ligands, Fisher's exact test *p*-value for enrichment of surface atoms in residues ranked by ET in £30 percentile within 5 Å of ligands, total number of surface atoms in residues ranked by Gupta et. al in £30 percentile, number of surface atoms in residues ranked by Gupta et. al in £30 percentile within 5 Å of ligands, Fisher's exact test *p*-value for enrichment of surface atoms in residues ranked by Gupta et. al in £30 percentile within 5 Å of ligands are listed.

Dataset S5. Structural Sites with 5A Proximity Cutoff. Structural sites for each of the proteins with solved structures. Sites reported have a size >= 2 residues, ET rankings <= 30, atoms within 5Å of one another, are surface accessible, and have no variants in the current outbreak. Sites are organized by protein, structure, and then site size.

Dataset S6. Linear Sites. Linear sites for each of the proteins in the SARS-CoV-2 proteome. The first page details all linear sites with regard to the reference genome, while the second page provides linear sites which map to the selected structures. Sites in this data set have a size >= 2 residues, ET rankings <= 30, are connected in sequence, and have no variants in the current outbreak. Sites are organized by protein, structure (for the second page), and site size.

Dataset S7. Comparison of Linear ET Sites and Structural ET Sites. Linear ET sites and structural ET sites were compared for each protein structure. Linear ET sites were truncated to match the coverage of the protein structure. The Jaccard similarity and Fisher exact test p-values were reported for each linear-structural site comparison.

Dataset S8. Cross-reactive T Cell Epitopes Training Set. For each SARS-CoV-2 homologous T-cell epitopes that were tested for cross reactive by Mateus et al., the percent identity, sumEA, sum(100-ET ranking), and sumEA/sum(100-ET ranking) were calculated. The optimal cutoff point for sumEA/sum(100-ET ranking) that best predict cross-reactivity was determined using this dataset.

Dataset S9. Cross-reactive T cell Epitopes Prediction Set. For each SARS-CoV-2 homologous T-cell epitopes that were not tested for cross reactive by Mateus et al., the sumEA/sum(100-ET ranking) were calculated. This metric (<=0.168) was then used to predict the cross-reactivity of these T-cell epitopes.

References

Alhammad,Y.M.O. *et al.* (2020) The SARS-CoV-2 conserved macrodomain is a highly efficient ADP-ribosylhydrolase. *bioRxiv*, 2020.05.11.089375.

Bateman,A. (2019) UniProt: A worldwide hub of protein knowledge. *Nucleic Acids Res.*, **47**, D506–D515.

Beigel,J.H. *et al.* (2020) Remdesivir for the Treatment of Covid-19 - Preliminary Report. *N. Engl. J. Med.*

Benson,D.A. *et al.* (2018) GenBank. *Nucleic Acids Res.*, **46**, D41–D47.

Bienert,S. *et al.* (2017) The SWISS-MODEL Repository-new features and functionality. *Nucleic Acids Res.*, **45**, D313–D319.

Camacho,C. *et al.* (2009) BLAST+: Architecture and applications. *BMC Bioinformatics*, **10**.

Chang,C. *et al.* (2020) RCSB PDB - 6VYO: Crystal structure of RNA binding domain of nucleocapsid phosphoprotein from SARS coronavirus 2.

Chen,J. *et al.* (2020) Structural Basis for Helicase-Polymerase Coupling in the SARS-CoV-2 Replication-Transcription Complex. *Cell*.

Clark,L.K. *et al.* (2020) Structure of Nonstructural Protein 1 from SARS-CoV-2. *J. Virol.*, **95**.

Decroly,E. *et al.* (2011) Crystal Structure and Functional Analysis of the SARS-Coronavirus RNA Cap 2′-O-Methyltransferase nsp10/nsp16 Complex. *PLoS Pathog.*, **7**, e1002059.

Gupta,R. *et al.* (2020) SARS-CoV-2 (COVID-19) structural and evolutionary dynamicome: Insights into functional evolution and human genomics. *J. Biol. Chem.*, **295**, 11742–11753.

Katsonis,P. and Lichtarge,O. (2014) A formal perturbation equation between genotype and phenotype determines the Evolutionary Action of protein-coding variations on fitness. *Genome Res.*, **24**, 2050–2058.

Kern,D.M. *et al.* (2020) Cryo-EM structure of the SARS-CoV-2 3a ion channel in lipid nanodiscs. *bioRxiv Prepr. Serv. Biol.*

Kim,Y. *et al.* (2020) RCSB PDB - 6WLC: Crystal Structure of NSP15 Endoribonuclease from SARS CoV-2 in the Complex with Uridine-5’-Monophosphate.

Kim,Youngchang *et al.* (2020) Tipiracil binds to uridine site and inhibits Nsp15 endoribonuclease NendoU from SARS-CoV-2. *bioRxiv*, 2020.06.26.173872.

Larkin,M.A. *et al.* (2007) Clustal W and Clustal X version 2.0. *Bioinformatics*, **23**, 2947–2948.

Li,H. (2011) A statistical framework for SNP calling, mutation discovery, association mapping and population genetical parameter estimation from sequencing data. *Bioinformatics*, **27**, 2987–93.

Li,H. (2018) Minimap2: Pairwise alignment for nucleotide sequences. *Bioinformatics*, **34**, 3094–3100.

Lichtarge,O. *et al.* (2002) Evolutionary traces of functional surfaces along G protein signaling pathway. In, *Methods in Enzymology*. Academic Press Inc., pp. 536–556.

Littler,D.R. *et al.* (2020) Crystal Structure of the SARS-CoV-2 Non-structural Protein 9, Nsp9. *iScience*, **23**.

Liu,B. *et al.* (2020) RCSB PDB - 6XQB: SARS-CoV-2 RdRp/RNA complex.

Lua,R.C. *et al.* (2016) UET: A database of evolutionarily-predicted functional determinants of protein sequences that cluster as functional sites in protein structures. *Nucleic Acids Res.*, **44**, D308–D312.

Lua,R.C. and Lichtarge,O. (2010) PyETV: A PyMOL evolutionary trace viewer to analyze functional site predictions in protein complexes. *Bioinformatics*, **26**, 2981–2982.

Ma,Y. *et al.* (2015) Structural basis and functional analysis of the SARS coronavirus nsp14-nsp10 complex. *Proc. Natl. Acad. Sci. U. S. A.*, **112**, 9436–9441.

Madabushi,S. *et al.* (2002) Structural clusters of evolutionary trace residues are statistically significant and common in proteins. *J. Mol. Biol.*, **316**, 139–154.

Mateus,J. *et al.* (2020) Selective and cross-reactive SARS-CoV-2 T cell epitopes in unexposed humans. *Science (80-.).*, eabd3871.

Mihalek,I. *et al.* (2004) A Family of Evolution-Entropy Hybrid Methods for Ranking Protein Residues by Importance. *J. Mol. Biol.*, **336**, 1265–1282.

Mihalek,I. *et al.* (2007) Background frequencies for residue variability estimates: BLOSUM revisited. *BMC Bioinformatics*, **8**.

Minasov,G., Shuvalova,L., Rosas-Lemus,M., Kiryukhina,O., Wiersum,G., *et al.* (2020) RCSB PDB - 6W4H: 1.80 Angstrom Resolution Crystal Structure of NSP16 - NSP10 Complex from SARS-CoV-2.

Minasov,G., Shuvalova,L., Rosas-Lemus,M., Kiryukhina,O., Satchell,K.J.F., *et al.* (2020) RCSB PDB - 6WKQ: 1.98 Angstrom Resolution Crystal Structure of NSP16-NSP10 Heterodimer from SARS-CoV-2 in Complex with Sinefungin.

Nelson,C.A. *et al.* (2020) RCSB PDB - 6W37: STRUCTURE OF THE SARS-CoV-2 ORF7A ENCODED ACCESSORY PROTEIN.

Newman,J.A. *et al.* (2020) RCSB PDB - 6ZSL: Crystal structure of the SARS-CoV-2 helicase at 1.94 Angstrom resolution.

Non-structural protein 4 (nsp4) | P0DTD1 PRO_0000449622 | Models.

Osipiuk,J. *et al.* (2020) Structure of papain-like protease from SARS-CoV-2 and its complexes with non-covalent inhibitors. *bioRxiv*, 2020.08.06.240192.

Owen,C.D. *et al.* (2020) RCSB PDB - 6YB7: SARS-CoV-2 main protease with unliganded active site (2019-nCoV, coronavirus disease 2019, COVID-19).

Rosas-Lemus,M. *et al.* (2020) The crystal structure of nsp10-nsp16 heterodimer from SARS-CoV-2 in complex with S-adenosylmethionine. *bioRxiv*, 2020.04.17.047498.

Rut,W. *et al.* (2020) Activity profiling and structures of inhibitor-bound SARS-CoV-2-PLpro protease provides a framework for anti-COVID-19 drug design. *bioRxiv Prepr. Serv. Biol.*

Shu,Y. and McCauley,J. (2017) GISAID: Global initiative on sharing all influenza data - from vision to reality. *Euro Surveill.*, **22**.

Studer,G. *et al.* (2020) QMEANDisCo-distance constraints applied on model quality estimation. *Bioinformatics*, **36**, 1765–1771.

Surya,W. *et al.* (2018) Structural model of the SARS coronavirus E channel in LMPG micelles. *Biochim. Biophys. Acta - Biomembr.*, **1860**, 1309–1317.

The PyMOL Molecular Graphics System.

Thoms,M. *et al.* (2020) Structural basis for translational shutdown and immune evasion by the Nsp1 protein of SARS-CoV-2. *Science (80-.).*, **369**, eabc8665.

Waterhouse,A. *et al.* (2018) SWISS-MODEL: Homology modelling of protein structures and complexes. *Nucleic Acids Res.*, **46**, W296–W303.

Wilkins,A.D. *et al.* (2013) Accounting for epistatic interactions improves the functional analysis of protein structures. *Bioinformatics*, **29**, 2714–2721.

Wilkins,A.D. *et al.* (2010) Sequence and structure continuity of evolutionary importance improves protein functional site discovery and annotation. *Protein Sci.*, **19**, 1296–1311.

Woo,H. *et al.* (2020) Developing a Fully Glycosylated Full-Length SARS-CoV-2 Spike Protein Model in a Viral Membrane. *J. Phys. Chem. B*, **124**, 7128–7137.

Wrapp,D. *et al.* (2020) Cryo-EM structure of the 2019-nCoV spike in the prefusion conformation. *Science*, **1263**, 1260–1263.

Yin,W. *et al.* (2020) Structural basis for inhibition of the RNA-dependent RNA polymerase from SARS-CoV-2 by remdesivir. *Science*, **368**, 1499–1504.

Zhang,L. *et al.* (2020) Crystal structure of SARS-CoV-2 main protease provides a basis for design of improved α-ketoamide inhibitors. *Science*, **412**, 409–412.

Zhao,W.-M. *et al.* (2020) The 2019 novel coronavirus resource. *Yi chuan = Hered.*, **42**, 212–221.

Zinzula,L. *et al.* (2020) RCSB PDB - 6ZCO: Crystal Structure of C-terminal Dimerization Domain of Nucleocapsid Phosphoprotein from SARS-CoV-2, crystal form II.
